# The predictive value of a concise classification of left atrial appendage morphology to thrombosis in non‐valvular atrial fibrillation patients

**DOI:** 10.1002/clc.23381

**Published:** 2020-05-14

**Authors:** Jionghong He, Zenan Fu, Long Yang, Wei Liu, Ye Tian, Qifang Liu, Zhi Jiang, Longhai Tian, Jing Huang, Shui Tian, Yidong Zhao

**Affiliations:** ^1^ Cardiology Department Guizhou Provincial People's Hospital Guiyang City China; ^2^ Department of Respiratory The Third Affiliated Hospital of Zunyi Medical University Zunyi City Guizhou Province China

**Keywords:** left atrial appendage, thrombosis, nonvalvular atrial fibrillation, risk factor

## Abstract

**Background:**

The complexity of left atrial appendage (LAA) in patients with nonvalvular atrial fibrillation (NVAF) is closely related to LAA thrombosis and stroke incidence. But the classification of LAA morphology is not uniform and controversial.

**Hypothesis:**

This study divided the LAA into two categories according to the LAA morphology to investigate the risk of thrombosis related to the LAA structural complexity in NVAF patients.

**Methods:**

A total of 336 NVAF patients were enrolled continuously in this study. The patients were divided into thrombosis group and non‐thrombosis group according to whether the thrombus presence in LAA. Through computer LAA three‐dimensional reconstruction, LAA morphology was divided into the complex type and simple type according to with or without the clearly lobulated structure judged by imaging experts. The relationship between LAA thrombosis and various potential risk factors was analyzed.

**Results:**

A total of 19 potential risk factors for LAA thrombosis in NVAF patients were enrolled into statistical analysis. The coincidence rate of LAA morphology classification was 96.4% (324/336) between two imaging experts. Multivariate logistic regression analysis showed that complex LAA morphology (OR 4.168, 95% CI 1.871‐9.288, *P* < .001) was associated with the presence of LAA thrombus, independently of other enrolled risks.

**Conclusions:**

It is a concise and reliable method to divide the LAA morphology into complex type and simple type according to whether with the clearly lobulated structure. The complex LAA is an independent risk factor for LAA thrombosis in NVAF patients.

## 1 INTRODUCTION

The prevalence of ischemic stroke in patients with atrial fibrillation (AF) is about five times of that in non‐AF patients.^1^ It is associated with a mortality rate of nearly 20% and a disability rate of nearly 60%.^2^ More than 90% of ischemic stroke thrombus in patients with non‐valvular atrial fibrillation (NVAF) was derived from the left atrial appendage (LAA).^3^, ^4^ Due to the special anatomical structure and functional characteristics of LAA, the thrombus is easy to form in the AF state. The complexity of LAA in patients with AF is closely related to LAA thrombosis and stroke incidence.^5^, ^6^, ^7^ At present, the classification of LAA morphology‐based classification method in the academic community is not uniform and controversial.^5^, ^6^, ^7^, ^8^ This study divided the LAA into two categories according to the LAA formation to investigate the risk of thrombosis related to the LAA structural complexity in patients with NVAF.

## 2 MATERIALS AND METHODS

### 2.1 Study patients

This retrospective study was approved by the Academic Committee of Guizhou Provincial People's Hospital, and all patients included had consented to the use of their data for research purposes.

A retrospective analysis was performed on NVAF patients who were undergoing radiofrequency ablation due to AF from January 2016 to April 2019. All patients were diagnosed with AF by conventional surface electrocardiogram or Holter examination, in line with “2014 AHA/ACC/HRS Guideline for the Management of Patients with Atrial Fibrillation.”^9^ The recommended level of AF catheter ablation is I, IIA, or IIB.^4^ Age and gender are not limited. Transesophageal echocardiography (TEE) and left atrial enhanced computed tomography angiography (CTA) were performed in all patients.

Exclusion Criteria: patients with valvular AF,^9^ acute coronary syndrome, acute stage of ischemic stroke, a history of hemorrhagic stroke, hyperthyroidism, hypothyroidism, severe hepatic/renal insufficiency, infectious disease, allergy to contrast media.

### 2.2 Instruments and equipment

A third‐generation force dual‐source CT (Siemens, Munich, Germany) was used, as was an IE33 echocardiography (Philips, Amsterdam, the Netherlands).

### 2.3 Patients data

#### 2.3.1 General data

Patients were recruited through our institution's Computerized Patient Record System. The following patient characteristics were documented upon participant recruitment: gender; age; type of AF; duration of AF (accurate to the month); oral anticoagulant use before admission; blood pressure during hospitalization; the presence of coronary heart disease, hypertension, diabetes, or vascular disease (old myocardial infarction, peripheral arterial disease, or aortic plaque); a history of stroke/transient ischemic attack (TIA)/thromboembolism (TE); and presenting either with or without symptoms and signs of congestive heart failure (CHF).

#### 2.3.2 Biochemical criterion

The following biochemical criteria were recorded: fasting serum glucose, brain natriuretic peptide (BNP), creatinine (Scr), plasma fibrinogen (Fbg) concentration, and prothrombin time international normalized ratio (INR) of every patient. All of which were measured for the first time after admission to the hospital.

#### 2.3.3 Imaging markers

First, patients with left atrial anterior‐posterior diameter (LAd), left ventricular end‐diastolic diameter (LVEDd), and left ventricular ejection fraction (LVEF) were measured by thoracic echocardiography. Moreover, chest X‐rays or computed tomography (CT) scans were used to determine whether aortic sclerosis was present. In addition, head CT or magnetic resonance imaging (MRI) was used to determine the presence or absence of cerebral infarction. Finally, coronary artery CT imaging or angiography was used to determine the degree of coronary artery stenosis, which aided in the diagnosis of coronary heart disease.

### 2.4 Clinical grouping

Based on the results of TEE and left atrial CTA, which were employed to assess whether a patient had a thrombus, the patients were subsequently divided into one of two groups: the thrombus group or non‐thrombus group. TEE is used as a “gold standard” for the diagnosis of LAA in patients with thrombus.^10^

### 2.5 Definition of indicators

#### 2.5.1 Classification of AF

According to the analysis requirements, AF was divided into two categories: paroxysmal AF and non‐paroxysmal AF (NPAF; including persistent AF, long‐range persistent AF, and permanent AF), which refer to the 2016 European society of cardiology (ESC) guidelines for the management of AF.^11^

#### 2.5.2 Definition of effective anticoagulation

The definition of effective anticoagulation is that the INR value of warfarin taken orally before TEE is ≥2.0 for at least 3 weeks, or when patients had taken the recommended dose of a new oral anticoagulant for at least 3 weeks.^11^

#### 2.5.3 ‐CHA_2_DS_2‐_ VASc model

The CHA_2_DS_2_‐VASc^10^ contains a total of eight risk factors: gender, age, CHF, hypertension, diabetes, coronary heart disease, stroke/TIA/TE, and vascular disease. For patients aged ≥75 years old and those with a history of previous stroke/TIA/TE, the score for each factor is two points. For patients between the ages of ≥65 years and < 75 years old, the remaining risk factors are given one point each.

#### 2.5.4 Acquisition and classification of LAA morphology

**FIGURE 1:**
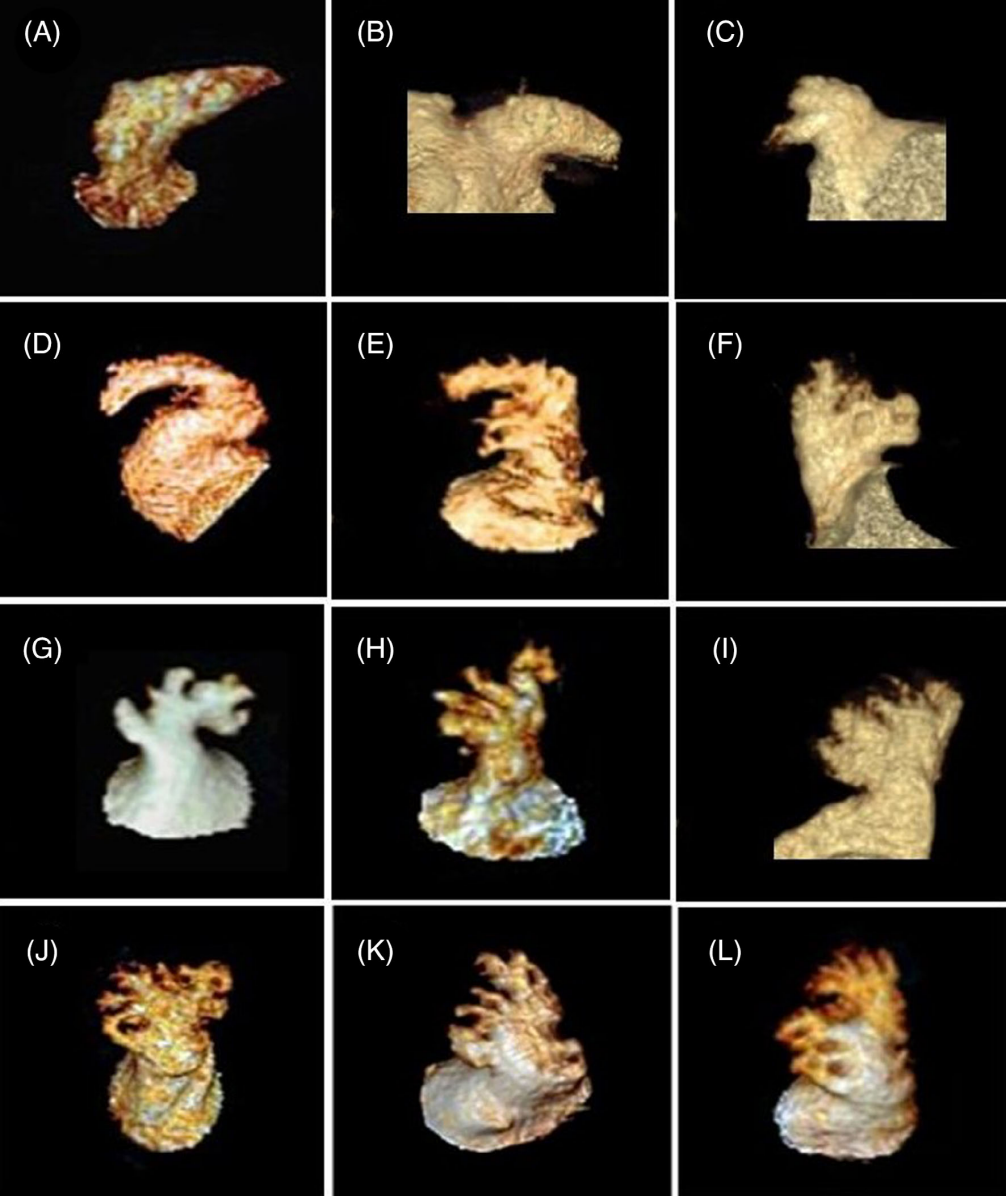
Various morphologies of the left atrial appendage. A‐D, simple left atrial appendage. E‐L, complex left atrial appendage

The three‐dimensional reconstruction of the left atrial CTA image was performed by CT image post‐processing system to obtain the LAA morphology of each patient; the image was classified based on the LAA's morphological characteristics. In this study, the LAA morphology was divided into two categories: simple‐LAA and complex‐LAA. Simple‐LAA was defined as only one backbone structure and no other distinct branch structures (Figure 1A‐D); a complex‐LAA featured with one or more distinct branching structure (Figure 1E‐L). Moreover, according to the way of Di Biase et al,^5^ the LAA morphology was classified into four categories, such as chicken wing‐like, wind bag‐like, cauliflower‐like, and cactus‐like. The morphology of LAA was determined by two imaging experts. When the results are inconsistent between the two imaging experts, the decision of another imaging expert is taken as the final result.

### 2.6 Statistical analysis

Statistical analysis was performed using SPSS 19.0 software. Each group of variables was tested for normality and homogeneity of variance. The measurement data conformed to be the normal distribution as mean ± SD; the univariate analysis used two independent sample *t tests*. If the measurement data did not conform to be the normal distribution, the median was used. The rank‐sum test was used for comparison between groups. Counting data use cases and percentage (%), the comparison between groups using *χ* ^*2*^ tests. Multivariate logistic regression analysis was used to investigate independent risk factors for LAA thrombosis. The raw data of AF course and BNP does not conform to the normal distribution, the natural logarithm transformation for these parameters were performed before logistic regression analysis.

## 3 RESULTS

### 3.1 Classification of LAA morphology

Among 336 patients, 162 (48.2%) cases had complex LAA morphology. The coincidence rate of LAA morphology classification was 96.4% (324/336) between two imaging experts, only 12 (3.6%) cases need the third expert to participate in the judgment.

According to Di Biase's LAA morphological categories,^5^ the proportion of chicken wing‐like (Figure 1A,B), wind bag‐like (Figure 1C‐G), cauliflower‐like (Figure 1H‐I) and cactus‐like (Figure 1 J‐L) LAA in this study were 46.1%, 12.8%, 27.1%, and 14.0%, respectively (Table 1). Among the four types LAA patients, the lowest incidence of LAA thrombus is the chicken wing‐like LAA group (9.0%), and that the highest is the cauliflower‐like LAA group (22.0%; Table 2). The incidence of LAA thrombus in chicken wing‐like LAA patients is markedly lower than that in non‐chicken wing‐like LAA patients (9.0% vs 17.7, *χ* ^2^ = 5.282，*P* = .022). In simple‐LAA, 89.1% of them were chicken wing‐like LAA, the remainder was wind bag‐like LAA. No cactus‐like and cauliflower‐like LAA belonged to simple‐LAA. The complex‐LAA included 14.8% of wind bag‐like, 56.2% of cauliflower‐like, and 29.0% of cactus‐like LAA, and without chicken wing‐like LAA. There were not statistically different to the proportion of LAA thrombus between simple‐LAA patients and chicken wing‐like LAA patients (9.2% vs 9.0%, *χ* ^2^ = 0.003，*P* = .959), as well as between complex‐LAA patients and non‐chicken wing‐like LAA patients (18.5% vs 17.7%, *χ* ^2^ = 0.041，*P* = .840).

**TABLE 1:** Comparison of the four forms of left atrial appendage (LAA) and LAA thrombus in simple‐LAA and complex‐LAA groups

	n (%)	Simple‐LAA (n [%])	Complex‐LAA (n [%])	*χ* ^2^	*P*
n (%)	336	174 (51.8)	162 (48.2)	—	—
Chicken wing	155 (46.1)	155 (89.1)	0 (0)	267.891	.000
Wind bag	43 (12.8)	19 (10.9)	24 (14.8)	1.141	.286
Cauliflower	91 (27.1)	0 (0)	91 (56.2)	134.044	.000
Cactus	47 (14.0)	0 (0)	47 (29.0)	58.691	.000
LAA thrombus	46 (13.7)	16 (9.2)	30 (18.5)	6.171	.013

**TABLE 2:** Comparison of the four forms of left atrial appendage (LAA) in thrombosis and non‐thrombosis groups

	n	Thrombosis	Non‐thrombosis	*χ2*	*P*
n (%)	336	46 (13.7)	290 (86.3)	—	—
Chicken wing (n [%])	155	14 (9.0)	141 (91.0)	5.284	.022
Wind bag (n [%])	43	5 (11.6)	38 (88.4)	0.178	.674
Cauliflower (n [%])	91	20 (22.0)	71 (78.0)	7.254	.007
Cactus (n [%])	47	7 (14.9)	40 (85.1)	0.067	.796
Non‐chicken wing (n [%])	181	32 (17.7)	149 (82.3)	5.284	.022

### 3.2 Comparison among groups

As shown in Table 3, complex‐LAA accounted for 65.2% and 45.5%, respectively, in thrombus group and non‐thrombus group (*P* = .013). Age (*P* < .002), AF course (*P* < .001), LAd (*P* < .001), and serum BNP concentration (*P* < .001) in the thrombus group were higher than those of patients in the non‐thrombus group, and the LVEF in the thrombus group was lower than that in the non‐thrombus group (*P* = .018). Compared with the non‐thrombus group, patients with thrombus had higher prevalence rates of male (*P* < .024), NPAF (*P* < .001), high‐risk CHA_2_DS_2_‐VASc score (*P* < .001), complex‐LAA (*P* < .001), hypertension (*P* = .025), diabetes mellitus (*P* < .001), coronary heart disease (*P* = .025), CHF (*P* < .001), stroke/TIA/TE, and vascular disease (*P* = .01). There were no significant differences in LVEDd, serum sCr concentration, plasma Fbg concentration, and effective anticoagulant rate between the two groups (all *P* > .05).

**TABLE 3:** Baseline characteristics

Variable name	Thrombosis group	Non‐thrombosis group	*t/Z/*_*χ2*_	*P*
n	46	290		
Age (years)	66.0 ± 9.9	60.8 ± 10.7	3.086	.002
Male gender	19 (41.3)	174 (60.0)	5.677	.024
AF course (mouth)	30 (1–180)	22 (1–120)	3.879	<.001
Non‐paroxysmal AF	35 (76.1)	59 (20.3)	61.224	<.001
high‐risk CHA_2_DS_2_‐VASc score	40 (87.0)	133 (45.9)	26.843	<.001
Complex LAA	30 (65.2)	132 (45.5)	6.171	.013
Hypertension	28 (60.9)	123 (42.4)	5.465	.025
Diabetes mellitus	13 (28.3)	23 (7.9)	17.153	<.001
Coronary artery disease	15 (32.6)	50 (17.2)	6.009	.025
Heart failure	17 (37.0)	12 (4.1)	54.225	<.001
Stroke/TIA/TE	12 (26.1)	25 (8.6)	12.360	.001
Vascular disease	38 (82.6)	148 (51.0)	16.016	<.001
Effective anticoagulation	11 (23.9)	59 (20.3)	0.306	.562
LAd(mm)	37.7 ± 7.0	33.8 ± 6.3	3.815	<.001
LVEDd(mm)	46.3 ± 5.6	45.6 ± 4.1	1.067	.287
LVEF(%)	57.4 ± 9.8	61.0 ± 6.2	2.446	.018
BNP(pg/mL)	288.7 (10.0‐915.5)	128.0 (10.0‐894.8)	6.060	<.001
Plasma fibrinogen (g/L)	2.75 ± 0.73	2.60 ± 0.67	1.335	.183
Serum creatinine (μmol/L)	83.9 ± 22.4	81.1 ± 21.9	0.790	.430

*Note*: Data are presented as mean ± SD, median (interquartile range), or n (%).

Abbreviations: AF, atrial fibrillation; BNP, brain natriuretic peptide; LAd, left atrial diameter; LAA, left atrial appendage; LVEDd, left ventricular end diastolic diameter; LVEF, left ventricular ejection fraction; TIA, transient ischemic attack; TE, thromboembolism.

### 3.3 Risk factors associated with thrombosis in LAA

Univariate analysis showed that there were three factors related to LAA thrombosis, such as AF course, LAd, NPAF, complex‐LAA, and LVEF (Table S1). With the three factors as independent variables and the presence or absence of thrombosis in LAA as the dependent variable, multivariate logistic regression analysis was conducted (Table 4). The results showed that complex‐LAA (OR 4.168, 95% CI 1.871‐9.288, *P* < .001), NPAF (OR 13.366, 95% CI 6.081‐29.380, *P* < .001), and AF course (OR 1.620, 95% CI 1.231‐2.131, *P* = .001) were the independent risk factors for the presence of LAA thrombosis.

**TABLE 4:** Results of multivariate logistic regression analysis

Variables						OR 95% CI	
	B	SE	Wald	*P*	OR	Lower limit	Upper limit
AF course	0.482	0.140	11.871	.001	1.620	1.231	2.131
Non‐paroxysmal AF	2.593	0.402	41.632	.000	13.366	6.081	29.380
Complex LAA	1.427	0.409	12.193	.000	4.168	1.871	9.288

Abbreviations: AF, atrial fibrillation; CI, confidence interval; LAA, left atrial appendage; OR, odds ratio.

## 4 DISCUSSION

The LAA is a long, curved, blind cavity‐like structure that extends from the anterior lateral wall of the left atrium and has many uneven trabeculars, which have active systolic and diastolic functions that play an important role in relieving internal pressure in the left atrium. The anatomical and functional characteristics of LAA are prone to thrombosis in the AF state. At present, there is no unified LAA morphology‐based classification method in the academic community. In 2012, Di Biase et al^5^ proposed that LAA morphology should be classified into four categories: chicken wing‐like, wind bag‐like, cactus‐like, and cauliflower‐like, as based on the structural characteristics of LAA in patients, as determined through cardiac CT or MRI images. In fact, LAA has many forms, and the classification of these four forms is not comprehensive. A variety of LAA forms are difficult to generalize (Figure 1). Moreover, Wu et al^8^ showed that, the classification of LAA morphology according to the four categories of Di Biase et al,^5^ a consensus among all three reviewers was only reached in 28.9% cases, so they believed that the classification of LAA by Di Biase et al^5^ is subjective, inaccurate, and unreliable.

This study divided the LAA into two categories (simple‐LAA and complex‐LAA), and compared with the four classifications of Di Biase et al.^5^ As shown in Table 1, the proportion of wind bag‐like LAA in simple‐LAA group and complex‐LAA group were 10.9% and 14.8%, respectively (*P* = .286). There were no cauliflower‐like and cactus‐like LAA in simple‐LAA group and no chicken wing‐like LAA in complex‐LAA group. The complexity of LAA in patients with AF is closely related to LAA thrombosis and stroke incidence.^5^, ^6^, ^7^ Di Biase et al.^5^ believed the chicken wing‐like LAA is the simplest structure, and the risk of stroke/TIA and LAA thrombosis is lower than other forms LAA. In this study, the simple‐LAA included not only chicken wing‐like LAA but also part of wind‐like LAA. So it raises a worry, our LAA classification approach is likely to divide high stroke risk patients into simple LAA group, and leading to clinical neglect of these patients. Our results showed the population of simple‐LAA group and chicken wing‐like LAA group were 174 and 155 patients respectively, and the proportion of LAA thrombus in the two groups was very close (9.2% vs 9.0%, *P* = .959). In this study, wind‐like LAA in simple‐LAA patients accorded with the characteristics of no obvious branches. The above results indicated that our LAA form classification is reasonable and helpful to distinguish the simple structure LAA and the complex structure LAA in wind bag‐like LAA patients.

Di Biase et al^5^ showed that the incidence of ischemic stroke/TIA was significantly lower in NVAF patients with the most morphologically simple form of LAA (chicken wing‐like) than in NVAF patients with the other three forms (wind bag‐like, cactus‐like, and cauliflower‐like) of LAA. In addition, Yamamoto et al^6^ found that the more LAA lobes in NVAF patients, the higher the proportion of LAA thrombosis. Both studies demonstrated that the more complex the LAA structure in NVAF patients, the greater the incidence of ischemic stroke/TIA or LAA thrombosis. This study divided the LAA into two categories (simple‐LAA and complex‐LAA), the consensus between two exports was reached in 96.4% cases, which may immensely reduce the subjective variability of LAA morphological classification.

We performed a statistical analysis of 19 potential risk factors for LAA thrombosis in NVAF patients and found that a total of 14 risk factors were statistically different between the thrombosis group and non‐thrombosis group (such as gender, age, complex‐LAA, AF course, NPAF, LAD, BNP, hypertension, diabetes, coronary heart disease, CHF, stroke/TIA/TE history, vascular disease, and high‐risk CHA2DS2‐VASc score), while the rate of effective anticoagulation, LVEDd, LVEF, and serum Fbg concentration were not statistically different between the two groups. Multivariate logistic regression analysis showed that complex LAA was an independent risk factor for presence of LAA thrombosis (OR 4.168, 95% CI 1.871‐9.288, *P* < .001).

In previous studies, cauliflower‐like LAA was regarded as the most complex LAA structure. This form of LAA has a short overall length, a large number of lobulated structures, and has been confirmed as an independent risk factor for stroke/TIA and LAA thrombosis by several studies.^5^, ^7^ In comparison, chicken wing‐like LAA is the simplest structure, and the risk of stroke/TIA and LAA thrombosis is low.^5^ If the analysis is based on the number of LAA lobes, then it can be said that the higher the number of lobes, the greater the proportion of LAA thrombosis.^6^ In this study, simple‐LAA was defined as an LAA with only one backbone structure and no other distinct lobulated structures; by contrast, complex‐LAA was defined as an LAA with one or more distinct lobulated structure. The simple‐LAA mainly includes chicken wing‐like LAA (Figure 1A‐B) and wind‐like LAA without other distinct lobulated structures (Figure 1C‐D), while other forms of LAA, including cactus‐like, cauliflower‐like, and wind‐like LAA with other distinct lobulated structures (Figure 1E‐G) are classified as complex‐LAA. The results of this study showed that the proportion of complex‐LAA in the thrombosis group was significantly higher than that in the non‐thrombosis group (65.2% vs 45.5%, *P* = .013). Moreover, complex‐LAA was an independent risk factor for LAA thrombosis (OR 4.168, 95% CI 1.871‐9.288, *P* < .001), which is consistent with the findings from previous studies.^6^

The reason why complex‐LAA is more likely to form a thrombus is related to LAA's lobulated structure, as well as its abundance of trabecular and lower LAA blood‐flow rate. Khurram et al^12^ found that cauliflower‐like LAA has extensive trabecular formation, and extensive LAA trabeculae are independently associated with a history of TIA/stroke in AF patients. LAA morphology is an independent determinant of LAA flow rate, and LAA flow rates in patients with chicken wing‐like LAA are significantly higher than those with cactus‐like or cauliflower‐like LAA.^13^ The caecum and lobulated structure of the LAA, as well as the uneven trabecular inside the LAA can slow down the blood‐flow rate and create a vortex, which may easily lead to blood deposition and thrombosis development. During AF attack, the active systolic and diastolic functions of LAA were inhibited, and the emptying of LAA was reduced, which further promoted the occurrence of hypercoagulable state and thrombosis.

The results of this study also showed that the course of AF among those patients in the thrombosis group was significantly longer than that in the non‐thrombosis group [30 (1‐180) months vs 22 (1‐120) months, *P* < .001), and the proportion of NPAF was significantly higher than that in the non‐thrombotic group (76.1% vs 20.3%, *P* < .01). The two factors were independent risk factors for thrombosis in LAA, which was consistent with the findings of previous studies.^14^, ^15^, ^16^

## 5 STUDY LIMITATIONS

In this study, of all 19 potential risk factors, only AF course, NPAF, and complex‐LAA were independent predictors of LAA thrombosis. However, several high‐risk independent risk factors of stroke proved by a large number of previous studies, such as heart failure, high‐risk CHA2DS2‐VASc score, and shock/TIA/TE history, were not risk factors of LAA thrombosis according to the results of this study. The main reason may be that the patients included in this study are limited to these with radiofrequency ablation indications (except for LAA thrombosis) in a single center, and only 336 patients enrolled, which cannot enough to represent the whole NVAF population.

## 6 CONCLUSIONS

In conclusion, it is a concise and reliable method to divide the LAA morphology into complex type and simple type according to with or without the clearly lobulated structure. The complex LAA is an independent risk factor for LAA thrombosis in NVAF patients.

## CONFLICT OF INTEREST

The authors declare no potential conflict of interests.

## Supporting information


**Table S1** Results of univariate analysis

**AppendixS1** Correction details
