# Supplementary material for: The predictive value of a concise classification of left atrial appendage morphology to thrombosis in non‐valvular atrial fibrillation patients
Source: Clin Cardiol. 2020 May 14;43(7):789–95. doi: 10.1002/clc.23381 (PMC7368353; doi:10.1002/clc.23381)
Supplement: Supplementary file 1 — Table S1 Results of univariate analysis [file CLC-43-789-s002.docx]

**Supplement table 1. Results of univariate analysis**

| variables | B | SE | Wald | P | OR | OR 95% CI | |
| --- | --- | --- | --- | --- | --- | --- | --- |
|  |  |  |  |  |  | Lower limit | Upper  limit |
| Age | 0.037 | 0.039 | 0.915 | .339 | 1.038 | 0.962 | 1.120 |
| Male gender | 1.064 | 0.602 | 3.119 | .077 | 2.897 | 0.890 | 9.436 |
| AF course | -.344 | 0.170 | 4.078 | .043 | 0.709 | 0.508 | 0.990 |
| Non-paroxysmal AF | 2.685 | 0.549 | 23.902 | .000 | 14.656 | 4.995 | 42.998 |
| High risk CHA_2_DS_2_-VASc score | 1.257 | 0.841 | 2.233 | .135 | 3.513 | 0.676 | 18.259 |
| Complex LAA | 1.011 | 0.491 | 4.238 | .040 | 2.748 | 1.050 | 7.193 |
| Coronary artery disease | 0.808 | 0.578 | 1.952 | .162 | 2.243 | 0.722 | 6.966 |
| Effective anticoagulation | 0.184 | 0.534 | 0.119 | .730 | 1.202 | 0.422 | 3.422 |
| Hypertension | 0.129 | 0.582 | 0.049 | .825 | 1.137 | 0.363 | 3.562 |
| Diabetes mellitus | 1.123 | 0.599 | 3.516 | .061 | 3.073 | 0.950 | 9.933 |
| Heart failure | 1.427 | 0.861 | 2.746 | .098 | 4.165 | 0.770 | 22.518 |
| Stroke/TIA/TE | 0.521 | 0.625 | 0.697 | .404 | 1.685 | 0.495 | 5.731 |
| Vascular disease | 0.449 | 0.800 | 0.315 | .575 | 1.567 | 0.327 | 7.512 |
| LAd | 0.080 | 0.043 | 3.442 | .064 | 1.083 | 0.995 | 1.179 |
| LVEDd | -0.009 | 0.054 | 0.027 | .868 | 0.991 | 0.892 | 1.102 |
| LVEF | 0.065 | 0.034 | 3.557 | .059 | 1.067 | 0.997 | 1.141 |
| BNP | -0.336 | 0.358 | 0.879 | .349 | 0.715 | 0.354 | 1.442 |
| Plasma fibrinogen | 0.506 | 0.390 | 1.686 | .194 | 1.658 | 0.773 | 3.559 |
| Serum creatinine | -0.009 | 0.012 | 0.564 | .453 | 0.991 | 0.967 | 1.015 |

AF, atrial fibrillation; LAA, left atrial appendage; BNP, brain natriuretic peptide; LAd, left atrial diameter; LVEDd, left ventricular end diastolic diameter; LVEF, left ventricular ejection fraction; TIA, transient ischemic attack; TE, thromboembolism.
