# Supplementary material for: The predictive value of a concise classification of left atrial appendage morphology to thrombosis in non‐valvular atrial fibrillation patients
Source: Clin Cardiol. 2020 May 14;43(7):789–95. doi: 10.1002/clc.23381 (PMC7368353; doi:10.1002/clc.23381)
Supplement: Supplementary file 2 — AppendixS1 Correction details [file CLC-43-789-s001.docx]

**Appendix S1.** Description of corrections.

*In Abstract, Results:*

Original:

**Results**: A total of 19 potential risk factors for LAA thrombosis in NVAF patients were enrolled into statistical analysis. The coincidence rate of LAA morphology classification was 96.4% (324/336) between two imaging experts. Multivariate logistic regression analysis showed that complex LAA morphology (OR 4.216, 95% CI 1.825-9.740, *P* = .001) was associated with the presence of LAA thrombus, independently of other enrolled risks.

Revised:

**Results**: A total of 19 potential risk factors for LAA thrombosis in NVAF patients were enrolled into statistical analysis. The coincidence rate of LAA morphology classification was 96.4% (324/336) between two imaging experts. Multivariate logistic regression analysis showed that complex LAA morphology (OR 4.168, 95% CI 1.871-9.288, *P* < .001) was associated with the presence of LAA thrombus, independently of other enrolled risks.

*In Section 3.3 “Risk factors associated with thrombosis in LAA”:*

Original:

Univariate analysis showed that there were five factors related to LAA thrombosis, such as AF course, LAd, NPAF, complex-LAA, and LVEF (Table S1). With the five factors as independent variables and the presence or absence of thrombosis in LAA as the dependent variable, multivariate logistic regression analysis was conducted (Table 4). The results showed that complex-LAA (OR 4.216, 95% CI 1.825-9.740, *P* = .001), NPAF (OR 12.326, 95% CI 5.506-27.593, *P* < .001), and AF course (OR 1931.569, 95% CI 55.049-67 774.874, *P* < .001) were the independent risk factors for the presence of LAA thrombosis.

Revised:

Univariate analysis showed that there were three factors related to LAA thrombosis, such as AF course, LAd, NPAF, complex-LAA, and LVEF (Table S1). With the three factors as independent variables and the presence or absence of thrombosis in LAA as the dependent variable, multivariate logistic regression analysis was conducted (Table 4). The results showed that complex-LAA (OR 4.168, 95% CI 1.871-9.288, *P* < .001), NPAF (OR 13.366, 95% CI 6.081-29.380, *P* < .001), and AF course (OR 1.620, 95% CI 1.231-2.131, *P* = .001) were the independent risk factors for the presence of LAA thrombosis.

*In Section 4 “Discussion,” paragraph 4, last sentence:*

Original:

Multivariate logistic regression analysis showed that complex LAA was an independent risk factor for presence of LAA thrombosis (OR 4.216, 95% CI 1.825-9.740, *P* = .001).

Revised:

Multivariate logistic regression analysis showed that complex LAA was an independent risk factor for presence of LAA thrombosis (OR 4.168, 95% CI 1.871-9.288, *P* < .001).

*In Section 4 “Discussion,” paragraph 5, last sentence:*

Original:

Moreover, complex-LAA was an independent risk factor for LAA thrombosis (OR 4.216, 95% CI 1.825-9.740, *P* = .001), which is consistent with the findings from previous studies.^6^

Revised:

Moreover, complex-LAA was an independent risk factor for LAA thrombosis (OR 4.168, 95% CI 1.871-9.288, *P* < .001), which is consistent with the findings from previous studies.^6^

*Table 4:*

Original:

Table 4. Results of multivariate logistic regression analysis

| Variables | B | SE | Wald | *P* | OR | OR 95% CI | |
| --- | --- | --- | --- | --- | --- | --- | --- |
|  |  |  |  |  |  | Lower limit | Upper limit |
| AF course | 7.566 | 1.815 | 17.372 | .000 | 1931.569 | 55.049 | 67 774.874 |
| Non-paroxysmal AF | 2.512 | 0.411 | 37.321 | .000 | 12.326 | 5.506 | 27.593 |
| Complex LAA | 1.439 | 0.427 | 11.346 | .001 | 4.216 | 1.825 | 9.740 |
| LVEF |  |  |  | .051 |  |  |  |
| LAd |  |  |  | .984 |  |  |  |

Abbreviations: AF, atrial fibrillation; CI, confidence interval; LAA, left atrial appendage; LAd, left atrial diameter; LVEF, left ventricular ejection fraction; OR, odds ratio.

Revised:

Table 4. Results of multivariate logistic regression analysis

| Variables | B | SE | Wald | *P* | OR | OR 95% CI | |
| --- | --- | --- | --- | --- | --- | --- | --- |
|  |  |  |  |  |  | Lower limit | Upper limit |
| AF course | 0.482 | 0.140 | 11.871 | .001 | 1.620 | 1.231 | 2.131 |
| Non-paroxysmal AF | 2.593 | 0.402 | 41.632 | .000 | 13.366 | 6.081 | 29.380 |
| Complex LAA | 1.427 | 0.409 | 12.193 | .000 | 4.168 | 1.871 | 9.288 |

Abbreviations: AF, atrial fibrillation; CI, confidence interval; LAA, left atrial appendage; OR, odds ratio.

*Table S1:*

Original:

**Supplement table. 1 Results of univariate analysis**

| variables | B | SE | Wald | P | OR | OR 95% CI | |
| --- | --- | --- | --- | --- | --- | --- | --- |
|  |  |  |  |  |  | Lower limit | Upper  limit |
| Age | 0.038 | 0.040 | 0.930 | 0.335 | 1.039 | 0.961 | 1.124 |
| Male gender | 0.980 | 0.605 | 2.627 | 0.105 | 2.665 | 0.814 | 8.721 |
| AF course | 6.217 | 2.444 | 6.474 | 0.011 | 501.346 | 4.170 | 60270.550 |
| Non-paroxysmal AF | 2.640 | 0.550 | 23.059 | 0.000 | 14.012 | 4.770 | 41.158 |
| High risk CHA_2_DS_2_-VASc score | 1**.26**2 | 0.863 | 2.142 | 0.143 | 3.534 | 0.652 | 19.162 |
| Complex LAA | 1.137 | 0.512 | 4.928 | 0.026 | 3.117 | 1.142 | 8.503 |
| Coronary artery disease | 0.778 | 0.584 | 1.776 | 0.183 | 2.177 | 0.693 | 6.835 |
| Effective anticoagulation | 0.243 | 0.549 | 0.196 | 0.658 | 1.275 | 0.435 | 3.735 |
| Hypertension | 0.200 | 0.591 | 0.115 | 0.735 | 1.222 | 0.383 | 3.893 |
| Diabetes mellitus | 1.094 | 0.610 | 3.211 | 0.073 | 2.985 | 0.902 | 9.877 |
| Heart failure | 0.362 | 0.639 | 0.321 | 0.571 | 1.437 | 0.410 | 5.030 |
| Stroke/TIA/TE | 0.564 | 0.820 | 0.473 | 0.491 | 1.758 | 0.352 | 8.764 |
| Vascular disease | 0.362 | 0.639 | 0.321 | 0.571 | 1.437 | 0.410 | 5.030 |
| LAd | 0.086 | 0.044 | 3.844 | 0.050 | 1.090 | 1.000 | 1.188 |
| LVEDd | -0.016 | 0.054 | 0.082 | 0.774 | 0.985 | 0.885 | 1.095 |
| LVEF | 0.071 | 0.035 | 4.110 | 0.043 | 1.074 | 1.002 | 1.150 |
| BNP | -0.242 | 0.274 | 0.779 | 0.377 | 0.785 | 0.458 | 1.344 |
| Plasma fibrinogen | 0.474 | 0.392 | 1.461 | 0.227 | 1.607 | 0.745 | 3.467 |
| Serum creatinine | -0.010 | 0.012 | 0.611 | 0.435 | 0.990 | 0.967 | 1.015 |

AF, atrial fibrillation; LAA, left atrial appendage; BNP, brain natriuretic peptide; LAd, left atrial diameter; LVEDd, left ventricular end diastolic diameter; LVEF, left ventricular ejection fraction; TIA, transient ischemic attack; TE, thromboembolism.

Revised:

Supplement table 1. Results of univariate analysis

| variables | B | SE | Wald | P | OR | OR 95% CI | |
| --- | --- | --- | --- | --- | --- | --- | --- |
|  |  |  |  |  |  | Lower limit | Upper  limit |
| Age | 0.037 | 0.039 | 0.915 | .339 | 1.038 | 0.962 | 1.120 |
| Male gender | 1.064 | 0.602 | 3.119 | .077 | 2.897 | 0.890 | 9.436 |
| AF course | -.344 | 0.170 | 4.078 | .043 | 0.709 | 0.508 | 0.990 |
| Non-paroxysmal AF | 2.685 | 0.549 | 23.902 | .000 | 14.656 | 4.995 | 42.998 |
| High risk CHA_2_DS_2_-VASc score | 1.257 | 0.841 | 2.233 | .135 | 3.513 | 0.676 | 18.259 |
| Complex LAA | 1.011 | 0.491 | 4.238 | .040 | 2.748 | 1.050 | 7.193 |
| Coronary artery disease | 0.808 | 0.578 | 1.952 | .162 | 2.243 | 0.722 | 6.966 |
| Effective anticoagulation | 0.184 | 0.534 | 0.119 | .730 | 1.202 | 0.422 | 3.422 |
| Hypertension | 0.129 | 0.582 | 0.049 | .825 | 1.137 | 0.363 | 3.562 |
| Diabetes mellitus | 1.123 | 0.599 | 3.516 | .061 | 3.073 | 0.950 | 9.933 |
| Heart failure | 1.427 | 0.861 | 2.746 | .098 | 4.165 | 0.770 | 22.518 |
| Stroke/TIA/TE | 0.521 | 0.625 | 0.697 | .404 | 1.685 | 0.495 | 5.731 |
| Vascular disease | 0.449 | 0.800 | 0.315 | .575 | 1.567 | 0.327 | 7.512 |
| LAd | 0.080 | 0.043 | 3.442 | .064 | 1.083 | 0.995 | 1.179 |
| LVEDd | -0.009 | 0.054 | 0.027 | .868 | 0.991 | 0.892 | 1.102 |
| LVEF | 0.065 | 0.034 | 3.557 | .059 | 1.067 | 0.997 | 1.141 |
| BNP | -0.336 | 0.358 | 0.879 | .349 | 0.715 | 0.354 | 1.442 |
| Plasma fibrinogen | 0.506 | 0.390 | 1.686 | .194 | 1.658 | 0.773 | 3.559 |
| Serum creatinine | -0.009 | 0.012 | 0.564 | .453 | 0.991 | 0.967 | 1.015 |

AF, atrial fibrillation; LAA, left atrial appendage; BNP, brain natriuretic peptide; LAd, left atrial diameter; LVEDd, left ventricular end diastolic diameter; LVEF, left ventricular ejection fraction; TIA, transient ischemic attack; TE, thromboembolism.
